# Clinical and Genetic Spectrum of Stargardt Disease in Argentinean Patients

**DOI:** 10.3389/fgene.2021.646058

**Published:** 2021-03-26

**Authors:** Marcela D. Mena, Angélica A. Moresco, Sofía H. Vidal, Diana Aguilar-Cortes, María G. Obregon, Adriana C. Fandiño, Juan M. Sendoya, Andrea S. Llera, Osvaldo L. Podhajcer

**Affiliations:** ^1^Laboratorio de Terapia Molecular y Celular (Genocan), Fundación Instituto Leloir, CONICET, Buenos Aires, Argentina; ^2^Servicio de Genética, Hospital de Pediatría Prof. Dr. Juan P. Garrahan, Buenos Aires, Argentina; ^3^Servicio de Oftalmología, Hospital de Pediatría Prof. Dr. Juan P. Garrahan, Buenos Aires, Argentina

**Keywords:** Stargardt disease, next-generation sequencing – NGS, Latin America, mutation, ABCA4 gene

## Abstract

**Purpose:**

To describe the clinical and molecular spectrum of Stargardt disease (STGD) in a cohort of Argentinean patients.

**Methods:**

This retrospective study included 132 subjects comprising 95 probands clinically diagnosed with STGD and relatives from 16 of them. Targeted next-generation sequencing of the coding and splicing regions of *ABCA4* and other phenocopying genes (*ELOVL4*, *PROM1*, and *CNGB3*) was performed in 97 STGD patients.

**Results:**

We found two or more disease-causing variants in the *ABCA4* gene in 69/95 (73%) probands, a single *ABC*A4 variant in 9/95 (9.5%) probands, and no *ABCA4* variants in 17/95 (18%) probands. The final analysis identified 173 variants in *ABCA4*. Seventy-nine *ABCA4* variants were unique, of which nine were novel. No significant findings were seen in the other evaluated genes.

**Conclusion:**

This study describes the phenotypic and genetic features of STGD1 in an Argentinean cohort. The mutations p.(Gly1961Glu) and p.(Arg1129Leu) were the most frequent, representing almost 20% of the mutated alleles. We also expanded the *ABCA4* mutational spectrum with nine novel disease-causing variants, of which eight might be associated with South American natives.

## Introduction

Stargardt disease (STGD), initially described by Karl Stargardt in 1909, is the most common hereditary macular dystrophy with an estimated prevalence of 1 in 8,000 to 1 in 10,000 people (Blacharski, 1988). Mutations in the adenosine triphosphate (ATP)-binding cassette A4 gene (*ABCA4*) are responsible for the autosomal recessive form of the disease (STGD1, OMIM: 248200) (Allikmets et al., 1997). Most STGD1 patients typically present with bilateral central visual loss in early adolescence and young adulthood with a progressive vision loss during their life (Garces et al., 2018). Ophthalmic examination classically reveals bilateral atrophic changes in the macula due to the loss of photoreceptors and the retinal pigment epithelium (RPE) cells, the presence of yellowish flecks at the level of the RPE, and frequent dark or silent choroid due to the accumulation of lipofuscin in the outer retinal layers (Zernant et al., 2011; Fujinami et al., 2015; Molday, 2015). However, *ABCA4*-associated retinopathies are clinically variable and genetically heterogeneous. Although age-onset and disease progression varies widely depending on the combination of specific disease-causing alleles and modifiers, early onset is associated with a worse prognosis, while late-onset is associated with a milder phenotype (Fujinami et al., 2015; Zernant et al., 2017; Cremers et al., 2020).

Even though there is a high allelic heterogeneity in *ABCA4* with more than 1200 disease-causing variants reported to date^1^, there are founder mutations associated with STGD1 in various racial and ethnic groups, especially in Europeans, African–Americans, and Asians (Cremers et al., 2020). Those mutations are scarcely found in the limited studies in Latin American STGD1 patients that showed additional founder mutations (Chacón-Camacho et al., 2013; Salles et al., 2018).

In the present study, we aimed to search for the first time the mutational spectrum in Argentinean Stargardt patients and to describe their clinical features. We also expanded the *ABCA4* mutational spectrum with nine novel disease-causing variants, of which eight might be associated with native South Americans. Furthermore, we found two *de novo* variants in *ABCA4*, including a novel one, c.6214A>C. This study represents an advance in the accurate diagnosis and genetic counseling of STGD1 patients in our country; we also show that disease-causing *ABCA4* variants prevalent in other Latin American countries such as Mexico and Brazil were seldom detected in our patients’ cohort.

## Materials and Methods

### Subjects and Clinical Evaluation

This retrospective study included 132 subjects comprising 82 probands and 16 families (16 probands and their relatives). Most subjects were from Argentina, except two from Uruguay. This study was conducted according to the Declaration of Helsinki. The protocol was approved by the Research and Ethics Committee of Hospital de Pediatría Prof. Dr. Juan P. Garrahan (HPG, protocol #892) and by the Bioethics Committee of Fundación Instituto Leloir (protocol #27). Written informed consent was obtained from all participants or legal guardians before enrollment in the study. Pre- and post-test counseling was offered at HPG for all participants. Self-referred ancestry and family history of retinal disorders were obtained from each participant. All the probands (*n* = 98) and two siblings suspected of STGD1 disease underwent a comprehensive ophthalmological evaluation between November 2015 and December 2017 at HPG. Clinical assessment included the best-corrected Snellen visual acuity (BCVA), converted to equivalent logMAR visual acuity, fundus examination, color fundus photography (RG), and autofluorescence imaging (AF) performed with a retinal fundus camera (Carl Zeiss Meditec, Germany). When feasible, fluorescein angiography (FA), electrophysiology tests (ERG), and optical coherence tomography (OCT) were evaluated. The fundus appearance was classified into four groups, and the AF pattern into three types (Fujinami et al., 2015). The age of onset and duration of the disease was assigned as described by Fujinami et al. (2015). Early onset of disease was defined as ≤ 17 years-old.

### DNA Sample Collection and Sequencing

Six ml of peripheral blood were collected into EDTA tubes from all probands; saliva samples were collected using Oragene buccal swabs kit (DNA Genotek) from family members. The samples were transported following strict standard operating procedures from HPG to Genocan at Fundación Instituto Leloir. Genomic DNA was extracted using the QIAamp DNA Mini Kit (Qiagen) and quantified in a Nanodrop 2000 spectrophotometer (Thermo Scientific).

Next-generation sequencing (NGS) was performed in probands (*n* = 95) and two siblings, using a custom panel created with the Ion AmpliSeq^TM^ Designer software (Thermo Fisher Scientific, Inc.). The panel allowed the analysis of coding exons and adjacent intronic sequences (±10 bp) of the *ABCA4* (NM_000350.2), *ELOVL4* (NM_022726.3), *PROM1* (NM_006017.2), and *CNGB3* (NM_019098.4) genes by sequencing of 130 amplicons distributed in 2 pools (pool 1: 66 amplicons; pool 2: 64 amplicons). The total panel size was 37.92 kb, with global coverage of 99.9%. DNA input was assessed with the Qubit fluorometer 2.0 (Thermo Fisher Scientific, Inc.) using the Qubit dsDNA HS (high-sensitivity) assay kit. Twenty nanograms of each sample (10 ng/pool) were used for library preparation with the Ion AmpliSeq^TM^ Library Kit 2.0 (Thermo Fisher Scientific, Inc.). Targeted sequencing was performed using the Ion Hi-Q Sequencing Kit (Thermo Fisher Scientific, Inc.) on the Ion PGM^TM^ platform (Ion Torrent; Thermo Fisher Scientific, Inc.). For segregation studies, family members’ samples were analyzed by Sanger sequencing to search for the variants detected in their respecting probands (Macrogen^2^).

### Bioinformatics Analysis

Primary data analyses comprising raw signal conversion, base calling, read filtering, and alignments to the hg19/GRCh37 human reference genome were performed using the Torrent Suite software v. 4.0 (Ion Torrent; Thermo Fisher Scientific, Inc.). Coverage analysis plug-in (5.0.2.0 version) was used to verify overall amplicon coverage and check for strand bias. Following primary data analysis, all BAM files were uploaded on the Ion Reporter Server Software (Thermo Fisher Scientific, Inc.) for variant calling and annotation, resulting in a table of 30–50 variants per patient. Further, the targeted region’s coverage was manually inspected with the Integrative Genomics Viewer (IGV^3^) and Alamut Visual v.2.9 (Interactive Biosoftware, Rouen, France).

Analysis of the target regions revealed the systematic failure of four amplicons (coverage < 20x), corresponding to exons 21 and 29 of the *ABCA4* gene, exon 3 of the *ELOVL4* gene, and exon 4 of the *PROM1* gene. Sanger sequencing was performed for these four exons and other regions identified with low coverage (<20x). Variants with minor allele frequency (MAF) > 1% reported in different population databases such as 1.000 Genomes Project^4^, dbSNP^5^ or the Genome Aggregation Database (gnomAD^6^) were initially filtered out. Causative ABCA4 variants with a high population frequency, such as the hypomorphic variant c.5603A > T p.(Asn1868Ile), were rescued by manually reviewing the filtered-out variants. Candidate variants were checked against ClinVar^7^ and Leiden Open Variation Database (LOVD; see footnote 1). Variants classified as benign or likely benign were excluded from the study. Variants not found in the databases mentioned above were subjected to an exhaustive PubMed^8^ literature search. The pathogenicity assessment was tested using the *in silico* prediction tools; Sorting Intolerant From Tolerant (SIFT^9^), Polymorphism Phenotyping v2 (PolyPhen-2^10^), Mutation Taster^11^, Human Splicing Finder 3.0^12^ and Combined Annotation Dependent Depletion (CADD^13^). Candidate variants were manually reviewed in the BAM file using Alamut Visual v.2.9 and validated using Sanger sequencing. Novel variants were classified using the terms “pathogenic,” “likely pathogenic,” “uncertain significance” (VUS), “likely benign,” and “benign,” according to the guideline of the American College of Medical Genetics and Genomics (ACMG) (Richards et al., 2015).

## Results

### Clinical Assessment of the Cohort

From the 100 patients recruited, including 98 probands and two siblings, three probands were excluded from the study after clinical reexamination (Supplementary Figure 1). The mean age at diagnosis of the remaining 95 probands was 18 years (range 3–48 years), and the average time from the age of onset to diagnosis was 5 years (range 0–44 years). NGS identified at least one *ABCA4* mutant allele in 78/95 (82%) probands (45 female and 33 male). At the time of enrollment, the mean age of the 78 probands was 34 years old (range 10–65 years), with a mean age at onset of 14 years old (range 4–47 years). Sixty-three probands (81%) presented early onset disease with a mean duration of the disease of 21 years (range 1–52 years). The patients’ cohort showed symmetric grade 3a or higher, fundus abnormalities consistent with central retinal atrophy and vision loss in both eyes with a mean BCVA of 1.2 logMAR (Snellen 20/300) showing a range of 0.1–2.3 logMAR (Snellen 20/25 to hand movements) in both eyes (Supplementary Table 1). Frequently, the vision loss was associated with photophobia and, in fewer cases, with slow dark adaptation. Besides, the mean foveal thickness was 136.5 μm (range 43–251 μm) in both eyes, and over half of the probands showed dark choroid on FA images (Supplementary Table 1). Of note was the fact that only 16 out of 82 patients with at least one variant found in *ABCA4*, displayed a previous family history of STGD.

### Variants Present in the *ABCA4* Gene and Their Correlation With Clinical Features

**FIGURE 1:**
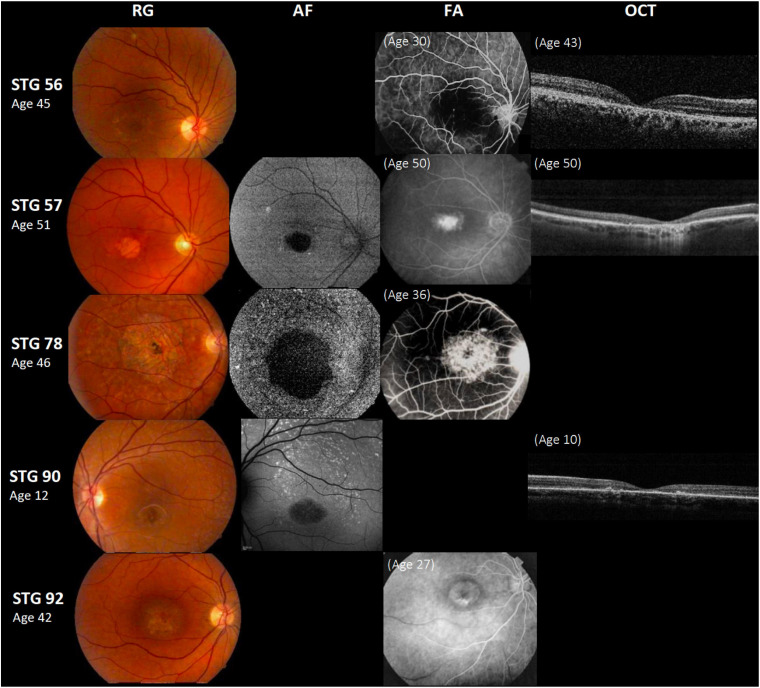
Clinical presentation in patients harboring a single *ABCA4* variant. Patients STG 56, STG 57, STG 78, and STG 90 show central atrophy surrounded by macular and peripheral flecks (fundus grade: 3b), which are more visible on AF image of STG 78 and STG 90, showing widespread multiple foci of high and low AF signal with heterogeneous background (AF type 2). These patients demonstrate dark “silent” choroid on Fluorescein angiography images (FA) and macular hyperfluorescence consequence of retinal pigment epithelium and photoreceptors loss. Patient STG 92 present on FA image at the age of 27 years-old, hyperfluorescent annular lesion showing perifoveal atrophy without flecks (Bull’s eye maculopathy) according to fundus grade 3a, further in the course of the disease with 42 years-old, note an enlargement of macular atrophy on color fundus photograph (RG). The optical coherence tomographic images (OCT) present decreased retinal thickness with disruption of external layers.

We found two variants or more in *ABCA4* in 69/95 (73%) probands (Supplementary Tables 2, 3); a single *ABCA4* variant was found in 9/95 (9.5%) probands with STGD1 phenotype. A second variant could be found in four of these nine patients by Khan et al. (2020) (Figure 1 and Supplementary Tables 2, 4). Therefore, a total of 73/95 (77%) probands of our cohort ended up having two or more *ABCA4* variants. In 17/95 (18%) probands, no variant was found in any of the sequenced genes.

The final analysis identified 173 variants in *ABCA4* spread out from exon 3 to exon 48. Seventy-nine variants were unique, and nine have not been previously associated with STGD (Table 1). Among the 79 unique variants found, the most common type of variant was missense (51/79), followed by frameshift (11/79), splice-site (9/79), nonsense (7/79), and in-frame deletion (1/79). All the variants were heterozygous, except for two homozygous variants identified in proband STG 80.

**TABLE 1:** Assessment of pathogenicity of novel *ABCA4* variants.

Patient ID	AO	rs#	chr. Pos.(GRCh37)	Ref	alt	g.DNA level	exon/intron	Nucleotide change (NM_000350.2)	Amino Acid Change (NP_000341.2)	Variant Effect	Polyphen- 2 (HumVar)	MT	SIFT	CADD (PHRED score)	HSF v3.0	Allele frequency% (gnomAD)	Source	Classification (ACMG)
STG 24	20	n.a	Chrl:94473812	A	T	g.94473812A>T	42	c.5877T > A	p. (Cys1959*)	Nonsense	n. a	Disease causing (prob: 1)	n.a	50	n.a	n.a	This study	Pathogenic
STG37	17	n.a	Chrl:94510299	AC	A	g.94510301del	20	c.2919de1	p.(leu973Phefs*4)	Frameshift	n. a	n. a	n.a	37	n.a	n.a	This study	Pathogenic
STG55	9	n.a	Chrl:94467482	T	G	g.94467482T>G	45	C.6214A>C	p.(Ser2072Arg)	Missense	Probably Damaging	Disease causing (prob: 1)	Deleterious (score: 0)	28.7	n.a	n.a	This study	Likely Pathogenic
STG58	15	n.a	Chrl:94577136	C	A	g. 94577136>A	12	c 161-1G>T	p.(?)	Splicing	n. a	Disease causing (prob: 1)	n.a	35	Alteration of the WT acceptor site, mos t probably affecting splicing.	n.a	This study	Pathogenic
STG82	20	n.a	Chrl:94502897	AT	A	g. 94502899del	25	c. 3617del	p. (Asn1206Metfs*3)	Frameshift	n. a	n. a	n.a	33	n.a	n.a	This study	Pathogenic
STG85	17	n.a	Chrl:94473236	C	A	g.94473236C>A	43	C.5959G > T	p.(Glyl987Trp)	Missense	Probably Damaging	Disease causing (prob: 1)	Deleterious (score: 0)	32	n.a	n.a	This study	Likely Pathogenic
STG86	8	n.a	Chrl:94502897	AC	A	g. 94502899del	25	c. 3617del	p. (Asn1206Metfs*3)	Frameshift	n. a	n. a	n.a	33	n.a	n.a	This study	Pathogenic
		n.a	Chrl:94480240	CG	C	g. 94480241del	38	c. 5318del	p.(Alal773Glyfs*5)	Frameshlft	n. a	n. a	n.a	33	n.a	n.a	This study	Pathogenic
STG99	47	rs7681 29542	chrl:94528730	G	T	g.94528730G > T	12	c. 1698C>A	p. (His566Gln)	Missense	Probably Damaging	Disease causing (prob: 0.999)	Deleterious (score: 0)	18.1	n.a	0.0007954	This study	VUS
		n.a	chrl:94528165	C	A	g.94528165C>A	13	c. 1905G>T	p. (Gln635His)	Missense	Probably Damaging	Disease causing (prob: 1)	Deleterious (score: 0)	24.7	n.a	n.a	This study	Likely Pathogenic

Eight probands carried nine heterozygous variants not previously associated with STGD. chr, chromosome; pos, position; ref, reference; alt, alteration; AO, age of onset; Polyphen- 2, polymorphism phenotyping v2; MT, mutation taster; CADD, combined annotation dependent depletion; SIFT, sorting intolerant from tolerant; HSF, human splicing finder; gnomAD, genome aggregation database; n.a, not available. The symbol “*” is part of the nomenclature (HGVS) to call the change at the protein level.

**FIGURE 2:**
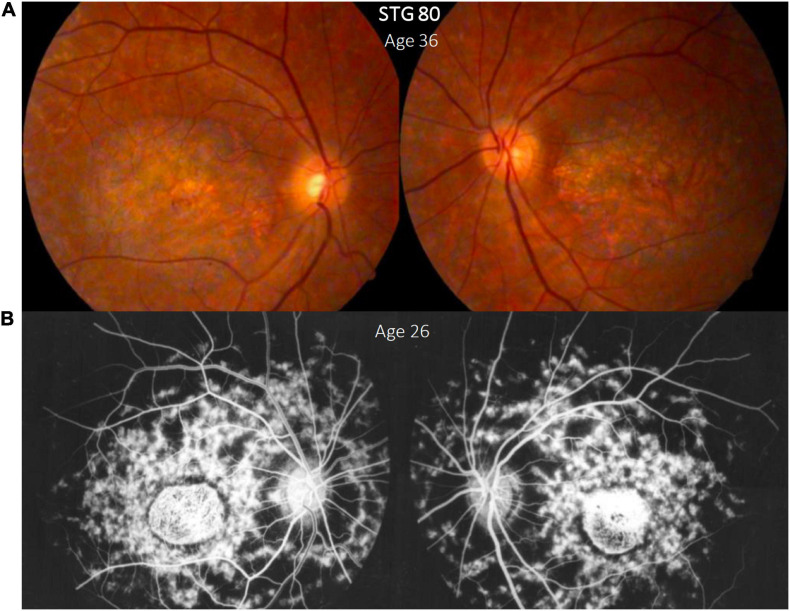
Clinical presentation in patient STG 80, **(A)** Color fundus photographs (RG) of both eyes with macular atrophy and peripheral flecks (fundus grade: 3b) **(B)** fluorescein angiography images (FA) clearly show dark “silent” choroid and central hyperfluorescence due to macular atrophy in this angiogram that was made years before our assessment. Note the enlargement of the diameter of the macular atrophy between the FA image brought by the patient **(B)** and the color RG **(A)** taken years later for this study.

Of the 156 alleles mutated in unrelated patients, p.(Gly1961Glu) and p.(Arg1129Leu) showed an allelic frequency of 10.25% (16/156) and 9.7% (15/156), respectively (Supplementary Figure 2). Half of the patients (8/16) harboring p.(Gly1961Glu) presented early onset of the disease, and 9/16 had severe visual impairment, with a mean BCVA of 1 logMAR for the right eye (range 0.2–2.3) and 0.9 logMAR for the left eye (0.2–1.9) and mean duration time of the disease of 24 years (2–50 years). Most patients (11/14) harboring p.(Arg1129Leu) demonstrated severe compromise in visual acuity with a mean BCVA of 1.2 logMAR (range 0.5–1.9) and a mean age of onset of 14 years old (range 7-24 years). Proband STG 80 carried two homozygous mutations p.(Arg1129Leu) and p.(Thr2240Ala) and showed a typical STGD phenotype (Figure 2). As expected, we observed that proband STG 9 and her affected brother (STG 10) carried the same variants; likewise, the monozygotic twins (STG 17 and STG 18) carried the same *ABCA4* variants; also, both pairs of siblings showed a concordant phenotype (Supplementary Table 2).

We performed segregation analysis in 16 families, although the co-segregation was not complete in six families due to the absence of additional family members (five cases) or incomplete sequencing information (one case) (Supplementary Figure 1).

We found the hypomorphic c.5603A>T, p.(Asn1868Ile) variant with an allelic frequency of 7% (13/190) mainly in heterozygous state and combined mostly with two causal *ABCA4* variants (Supplementary Tables 2, 3), except two probands who carried one (STG 2) or no variant (STG 41). STG 2 carried a heterozygous frameshift variant combined with c.5603A>T; upon segregation analysis, we found that both parents harbored c.5603A>T in heterozygous state.

#### Complex Alleles in *ABCA4* and Their Clinical Correlates

We detected complex *ABCA4* alleles in 13.7% (13/95) probands (Supplementary Table 3). Most of these probands carried variants previously identified as complex *ABCA4* alleles in retinal dystrophy patients. All but one patient (STG 15) presented early onset, and their clinical records were associated with higher aggressive disease, severe visual loss, and early morphologic changes (Supplementary Table 3).

The frequent complex allele, c.[1622T>C;3113C>T], p.[Leu541Pro;Ala1038Val] was found in two probands (STG 15 and STG 42), that also carried p.(Gly1961Glu). This complex allele was associated with a complete *ABCA4* loss of function and early onset, resembling truncating *ABCA4* variants (Zhang et al., 2015; Cremers et al., 2020). However, STG 15 had a late-onset at 24 years old, legal blindness after 20 years of the disease, showed macular atrophy with flecks, and compromised OCT images, likely due to the mild mutation p.(Gly1961Glu) present in the second allele; on the other side, STG 42, had an early onset at 16 years-old; this patient, that was enrolled 2 years after disease onset, showed a moderate visual loss and fundus with foveal atrophy without flecks. It is likely that a long-time follow-up would clarify the severity of the disease and the clinical correlation with this specific genotype in both patients (Supplementary Table 3). Segregation analysis confirmed the presence of the complex *ABCA4* alleles in relatives of STG 14, STG 15, and STG 42 (Supplementary Table 3 and Supplementary Figure 1).

STG 81 presented the complex allele c.688T>A(;)6718A>G. Of note, the variant c.6718A>G was not present in STG 81 family, even when the other two variants that were part of the genotype, c.688T>A and c.5714+5G>A were present (Supplementary Table 3). Therefore, we considered c.6718A>G as a *de novo* variant.

Five probands carried the hypomorphic variant c.5603A>T combined with the disease-causing variants c.5461-10T>C, 4469G>A p.(Cys1490Tyr), and c.2588G>C p.(Gly863Ala) (Supplementary Table 3). Segregation analysis in these five probands was not possible due to the lack of DNA samples of their family members. Other probands harboring c.5603A>T combined with two *ABCA4* variants not included as complex alleles are also shown (Supplementary Table 2).

#### Novel Variants and Correlation With the Clinics

**FIGURE 3:**
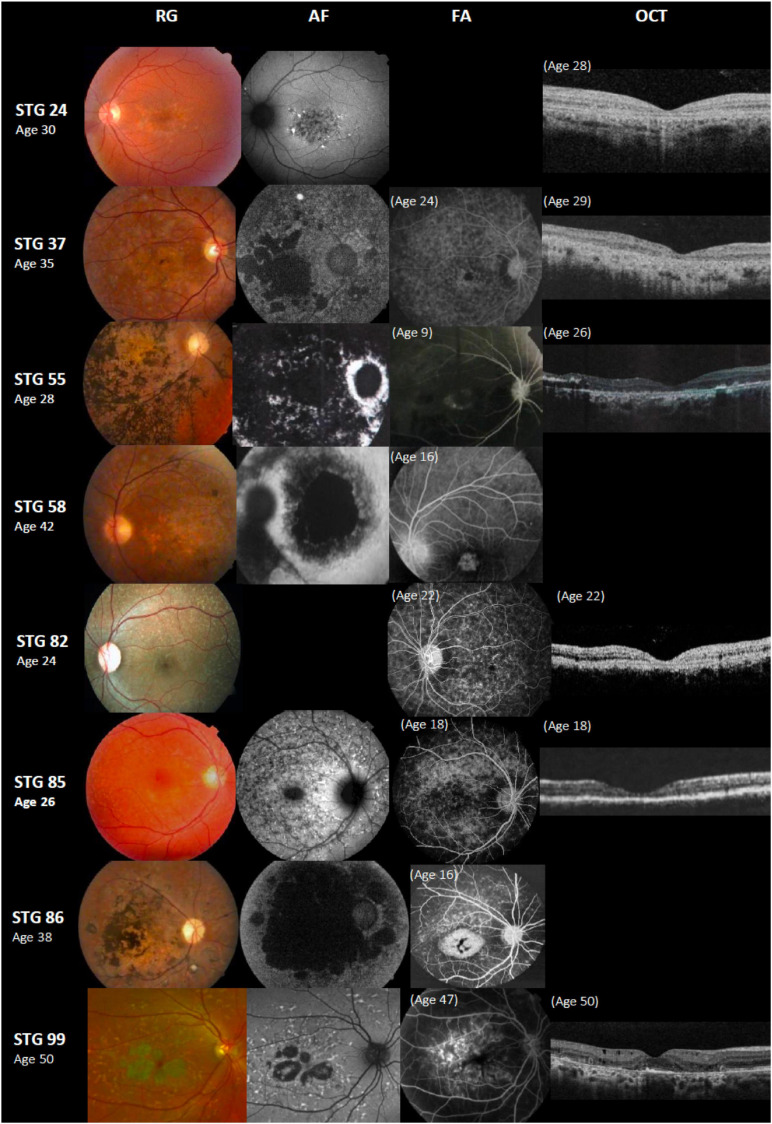
Color fundus photographs (RG), autofluorescence (AF), fluorescein angiography (FA), and optical coherence tomographic images (OCT) of eight patients with novel *ABCA4* variants. All of the shown cases had early onset except STG 24, STG 82, and STG 99. Patients STG 24, STG 37, STG 55, STG 82, and STG 85 present central atrophy with macular and peripheral flecks (fundus grade: 3b) on RG images; AF images demonstrate low AF signal at the macula and posterior pole as black lesions corresponding to cells death of retinal pigment epithelium and photoreceptors. The available OCT images in these patients present severe decreased retinal thickness below 150 μm and absence of external retinal layers. Patient STG 55 presents on FA image at the age of 9 years-old, hyperfluorescent annular lesion showing perifoveal atrophy without flecks (Bull’s eye maculopathy). Further, in the course of the disease with 28 years-old, note on AF how retinal atrophy increases as multiple areas of low AF signal at the posterior pole (AF type 3) and on RG image, there is extensive chorioretinal atrophy with large bone spicule pigmentation. Patient STG 58 at the age of 16 years-old, showed hyperfluorescent foveal lesion compatible with central atrophy on the FA image, that 26 years later in RG and AF images showed multiple extensive atrophic changes of the retinal pigment epithelium extending beyond the vascular arcades (fundus grade:4, AF type 3). Patient STG 86 showed on FA image at the age of 16 years-old, hyperfluorescent central lesion as macular atrophy surrounded by hyperfluorescent flecks. RG and AF images performed 22 years later, and there is an enlargement of macular atrophy with pigmentation clearly seen on RG and multiple areas of low AF signal in the posterior pole (AF type 3). Patient STG 99 presents central atrophy with foveal sparing and peripheral flecks in the color image; AF demonstrates low central AF signal with foveal sparing surrounded by marked flecks corresponding to foci of high signal as lipofuscin deposits. This patient had late onset of the disease at the age of 47 years old with BCVA 0.1 logMAR (20/25 Snellen) in both eyes. OCT image showed parafoveal disruption of external retinal layers with chronic structural changes.

**FIGURE 4:**
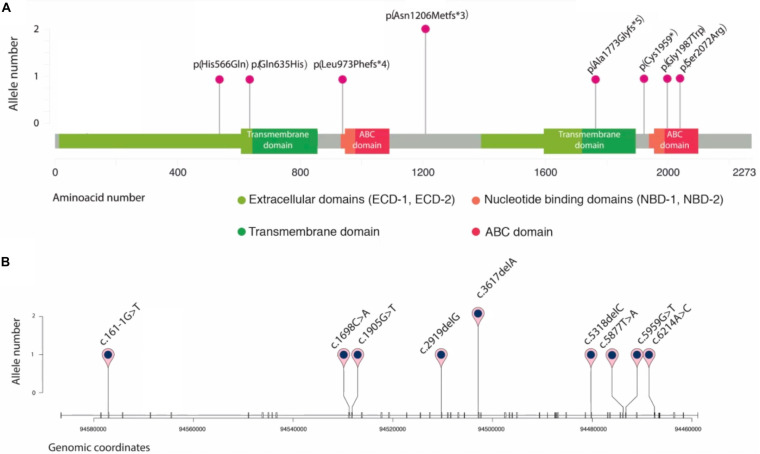
Schematic representation of novel *ABCA4* variants found in eight unrelated patients. Plots are adapted from cBioportal’s MutationMapper and R package trackViewer. Variants are displayed in two contexts: **(A)**: linear ABCA4 protein sequence (NP_000341.2), highlighting its typical A-subclass ABC transporter domains, and **(B)**: *ABCA4* gene sequence (NM_00350.2), where each tick represents an exon. Genomic coordinates correspond to hg19/GRCh37 human reference genome.

We found nine *ABCA4* novel variants in eight unrelated patients with STGD clinical phenotypes (Figure 3); eight were considered as likely pathogenic/pathogenic, and one as VUS (Table 1). Four were missense, three frameshifts, one nonsense, and one splice-site variant (Figure 4). None but one variant (c.1698C>A) has been previously found in two alleles in the population database gnomAD (Table 1). Most of the probands (5/8) carried novel variants associated with a known *ABCA4* mutation and exhibited clinical features of STGD1 with early onset (Supplementary Table 2).

Interestingly, STG 86, a 38-year-old female, carried two novel frameshifts variants p.[(Ala1773Glyfs^∗^5);(Asn1206Metfs^∗^3) that were considered pathogenic. She presented early onset with a visual acuity markedly affected (1.9 logMAR) after 30 years of the disease and a typical STGD atrophic fundus appearance (Supplementary Table 2 and Figure 3). Segregation analyses showed that both unaffected parents were heterozygous carriers of each one of those frameshift variants (Supplementary Figure 1).

Patient STG 99, a 50-year-old female, carried two novel missense variants p.(His566Gln)(;)(Gln635His). This patient presented late-onset (47 years old) with preserved visual acuity [BCVA of 0.1 logMAR (20/25 Snellen)] combined with paracentral atrophy with foveal sparing, peripheral flecks, and dark choroid on AF images consistent with STGD features (Figure 3). We were unable to perform segregation analysis due to the lack of DNA samples of her family. Of note, a different variant in the same amino acid position p.(Gln635Lys) was reported in a patient with STGD1 (Rivera et al., 2000) and classified as likely pathogenic (ClinVar: VCV000099095.2).

Patient STG 85 carried three variants; two known variants and a novel one, c.5959G>T, that we could confirm as present in *cis* with c.5961_5964del, as they both appeared in the same read in the NGS BAM file. Segregation analysis showed that the unaffected mother was heterozygous carrier of c.5714+5G>A (Supplementary Table 3 and Figure 3).

Interestingly, in patient STG 55 (Figure 3), the novel variant c.6214A>C p.(Ser2072Arg) was absent in either of the parents; therefore, we considered it as a *de novo* variant.

### Variants Present in *PROM1*, *ELOVL4*, and *CNBG3* Genes

Sequencing of the *PROM1* and *ELOVL4* genes showed only variants classified as benign or likely benign in all probands. A novel heterozygous variant, c.1528C>T p.(Leu510Phe), was detected in the *CNGB3* gene in proband STG 52. *In silico* analysis predicted p.(Leu510Phe) as deleterious on protein function [Polyphen-2 (score: 0.890), Mutation Taster (*p*-value: 0.989), and SIFT (score: 0.03)]. Patient STG 52 also carried c.1957C>T, p.(Arg653Cys) and c.6320G>A p.(Arg2107His) in the ABCA4 gene. This patient presented early onset disease with a slow progression in vision loss after 19 years of the disease (0,6 logMAR; 20/80 Snellen). Considering all evidence, p.(Leu510Phe) was classified as VUS.

## Discussion

In the present study, we performed the first clinical and molecular screening of STGD in Argentinean patients using targeted next-generation sequencing. This study revealed a total of 173 variants identified in *ABCA4*, from which 79 were unique. More than half were detected only once. Among them, we found nine novel variants. The most common *ABCA4* mutations were c.5882G>A p.(Gly1961Glu) and c.3386G>T p.(Arg1129Leu), representing almost 20% of the mutated alleles found in this representative cohort.

The mutation p.(Gly1961Glu) is the most common among STGD1 patients from different ethnic backgrounds and was reported with variable frequency (6.5–21%) (Ścieżyńska et al., 2016; Fujinami et al., 2018). Distinct clinical phenotypes were associated with patients harboring p.(Gly1961Glu) (Cella et al., 2009; Burke et al., 2012), including late-onset disease, Bull’s eye maculopathy, and the absence of dark choroid signs on FA and retinal flecks (Cella et al., 2009; Lee et al., 2017). Our patients harboring p.(Gly1961Glu) presented a mean age of onset disease of 18 years old, but only 1/16 probands exhibited Bull’s eye maculopathy. Furthermore, most of these probands presented flecks on RG, and dark choroid on FA, showing differences in the clinical phenotype of our STGD1 patients carrying this mutation compared to patients from other ethnic backgrounds (Cella et al., 2009; Lee et al., 2017).

The second more frequent pathogenic variant in our cohort, p.(Arg1129Leu), is considered a mild variant associated with later onset and foveal sparing (Del Pozo-Valero et al., 2020) that might enhance disease severity when combined with a “null mutation” (Riveiro-Alvarez et al., 2013). Interestingly, most of our patients harboring p.(Arg1129Leu) presented early onset (range 7–24 years) with a phenotype that included visual acuity already compromised at early stages, regardless of whether it was combined with a “null” or missense variant; this suggests once again that the *ABCA4* variants alone might not predict neither onset nor severity. One of these patients was double homozygous for p.(Arg1129Leu) combined with p.(Thr2240Ala). A few rare cases of double homozygous mutations were described in STGD1 patients (Burke et al., 2012; Serapinas et al., 2013). Unfortunately, no segregation analysis could be performed on this patient. Further studies, such as copy number variation and haplotype analysis, should be performed to exclude uniparental disomy (UPD) as previously reported in STGD1 patients (Fingert et al., 2006; Riveiro-Alvarez et al., 2007; Khan et al., 2020).

We found the hypomorphic c.5603A>T p.(Asn1868Ile) variant with an allele frequency of ∼7%, three times higher than in the general Latin American population (MAF ∼2.28) (see footnote 6). This result is consistent with previous studies showing that c.5603A>T (p.Asn1868Ile) is present three to four times more frequently in *ABCA4*-associated retinopathy patients than in the general population (Aguirre-Lamban et al., 2011; Zernant et al., 2017). Besides, c.5603A>T was found in probands that carried the disease-causing alleles c.5461–10T>C, p.(Cys1490Tyr), and p.(Gly863Ala). The variant c.5603A>T was reported to be always in *cis* with these alleles, and it was shown to be required for the pathogenicity of p.(Gly863Ala) (Zernant et al., 2017; Midgley et al., 2020).

It was of note the near absence of the Latin American-exclusive mutations described in the Mexican and Brazilian populations. Indeed, the most prevalent mutation in the Mexican population p.(Ala1773Val) (Chacón-Camacho et al., 2013) was utterly absent in our cohort; moreover, p.(Arg602Trp), the most prevalent mutation in Brazilian patients (Salles et al., 2018) was only found in one allele (0.6%). Besides, we found that only ∼14% of our probands harbored complex alleles in *ABCA4* compared to 30% reported in Brazilian patients (Salles et al., 2017). These findings can be explained by Argentina’s differential ancestral admixture compared to Brazil and Mexico (Muzzio et al., 2018). In that sense, p.(Arg1129Leu), a founder mutation in Spain (Valverde et al., 2006) considered rare in many populations, was found in 9.62% (15/156) alleles in our cohort. Also, two probands (STG14 and STG 27) carried the same set of three *ABCA4* variants that were reported in patients from a Spanish cohort (Riveiro-Alvarez et al., 2013). Further rare variants such as p.(Leu2140Gln); p.(Ser84Thrfs^∗^16) and p.(Leu464Glufs^∗^2) reported either as novel variants or only found in Italian cohorts (Simonelli et al., 2000; Fumagalli et al., 2001; Passerini et al., 2010; Testa et al., 2012) were identified in our cohort. In fact, patients harboring these mutations self-referred with admixture ancestry, mostly from Spain and Italy, consistent with the immigration history of Argentina.

Founder mutations in Danish and Chinese patients such as p.(Asn965Ser) (Rosenberg et al., 2007; Jiang et al., 2016; Cremers et al., 2020) or p.[Gly863Ala,Gly863del] in Western/Northern Europe (Maugeri et al., 1999, 2002) were detected at low frequency in our cohort. Further, the most frequent complex allele p.[Leu541Pro; Ala1038Val] described as a founder mutation in Germany (Rivera et al., 2000) also reported in high-frequency in patients from Eastern Europe (Ścieżyńska et al., 2016; Zolnikova et al., 2017; Tracewska et al., 2019) was found in 1.3% of our mutated alleles.

Eight unrelated patients carried nine novel variants. Two probands carried two novel variants each, while in the other cases, the novel variant was accompanied by known disease-causing *ABCA4* variants. Interestingly, two unrelated patients carried the same novel frameshift variant, p.(Asn1206Metfs^∗^3). All the probands with novel variants showed typical STGD1 phenotype, including the proband with two novel missenses, who showed late-onset and foveal sparing, apparently presenting the milder end of the clinical spectrum STGD1 (van Huet et al., 2014). Thus, it is tempting to hypothesize that eight of these novel variants might be associated with Native Americans from South America.

A study performed on 27 probands of our cohort with a single or no disease-causing *ABCA4* variants using single-molecule molecular inversion probes (smMIPs) led to the identification of the second coding/splice site *ABCA4* variant in three probands and two deep intronic variants in one proband (Khan et al., 2020); the three coding/splice site variants were confirmed in our NGS BAM files; however, they were missed in the analysis using Ion reporter variant caller software. These results indicate the necessity to combine and normalize the output of various variant calling tools to improve the sensitivity and precision to identify real variants (Sandmann et al., 2017).

Overall, a total of six probands could not be genetically solved, including five probands with a single *ABCA4* variant and a proband (STG 2) harboring a frameshift variant and the hypomorphic variant c.5603A>T in heterozygous state. This latter variant is considered a mild conditional allele, causal of STGD1 when it is present in *trans* with a loss-of-function variant (Zernant et al., 2017). In this regard, the segregation analysis within the STG 2 family could not establish the variants’ configuration (*cis/trans*) because both parents were heterozygous carriers of the c.5603A>T variant. Further studies involving haplotype analysis should be performed to establish the phase of both variants. No other *ABCA4* disease-causing variant was found in this proband when tested by Khan et al. (2020).

The missing *ABCA4* disease-causing alleles in these five probands could result from changes that affect *ABCA4* transcription or heterozygous deletions (<400 bp), insertions, or inversions (>40 bp), not detected by smMIPs-based sequencing (Khan et al., 2020). Although all our patients were diagnosed clinically with STGD, the overlapping of phenotypes between different macular dystrophies and several stages of *ABCA4*-associated retinopathy could result in a misdiagnosis of STGD with mutations in phenocopying genes, such as *PRPH2*, *ROM1, CRX*, or *RPGR* (Cremers et al., 2020). Other genomic techniques, including whole-exome sequencing, might eventually help to determine the molecular diagnosis of the remaining unsolved patients.

This study represents the first integrative study of retinopathy in Argentina. We are aware of the limitations of the study, in particular, the segregation studies due to the fact that families were difficult to approach and, in some cases, were reluctant to be included. Despite that, we can conclude that p.(Gly1961Glu) and p.(Arg1129Leu) were the most frequent mutations in our cohort, consistent with a Southern European ancestry’s broad representation in Argentina population’s admixture. We also expanded the *ABCA4* mutational spectrum with nine novel disease-causing variants; eight of them might be associated with native South Americans. Interestingly, there were phenotypic differences in patients of this cohort harboring mutations already described in the literature compared to other cohorts. These findings, along with the lack of concordance with variants seen in other Latin American populations, reinforce the need for genotype-phenotype studies in large cohorts and diverse populations.

## Data Availability Statement

The datasets presented in this study can be found in the online repository ABCA4 LOVD (https://databases.lovd.nl/shared/genes/ABCA4). The names of the repository/repositories and accession number(s) can be found below https://databases.lovd.nl/shared/variants?search_owned_by_=%3D%22Marcela%20Mena%22.

## Ethics Statement

This protocol was approved by the Research and Ethics Committee of Hospital de Pediatría Prof. Dr. Juan P. Garrahan (HPG, protocol #892) and by the Bioethics Committee of Fundación Instituto Leloir (protocol #27). Written informed consent to participate in this study was provided by the participants or legal guardians.

## Author Contributions

MM, AL, and OP contributed to conception and design of the study. AM and MO recruited the patients and performed the genetic counseling. SV and AF performed the clinical examination. MM and JS performed the NGS sequencing. JS, DA-C, and MM performed the Sanger sequencing. MM analyzed and interpreted the sequencing data, wrote the original draft of the manuscript. SV and AM wrote sections of the manuscript. MM, AL, and OP wrote, reviewed, and edited the manuscript. All the authors have read and agreed to the submitted version of the manuscript.

## Conflict of Interest

The authors declare that the research was conducted in the absence of any commercial or financial relationships that could be construed as a potential conflict of interest.

## Footnotes

1 https://www.lovd.nl

2 http://www.macrogen.com/

3 www.broadinstitute.org/igv

4 https://www.ncbi.nlm.nih.gov/variation/tools/1000genomes/

5 https://www.ncbi.nlm.nih.gov/snp/

6 http://gnomad.broadinstitute.org/

7 https://www.ncbi.nlm.nih.gov/clinvar/

8 https://pubmed.ncbi.nlm.nih.gov/

9 http://sift.jcvi.org/

10 http://genetics.bwh.harvard.edu/pph2/

11 http://www.mutationtaster.org/

12 http://www.umd.be/HSF/

13 https://cadd.gs.washington.edu/
