# Supplementary material for: Clinical and Genetic Spectrum of Stargardt Disease in Argentinean Patients
Source: Front Genet. 2021 Mar 26;12:646058. doi: 10.3389/fgene.2021.646058 (PMC8033171; doi:10.3389/fgene.2021.646058)
Supplement: Supplementary file 1 [file Data_Sheet_1.PDF]

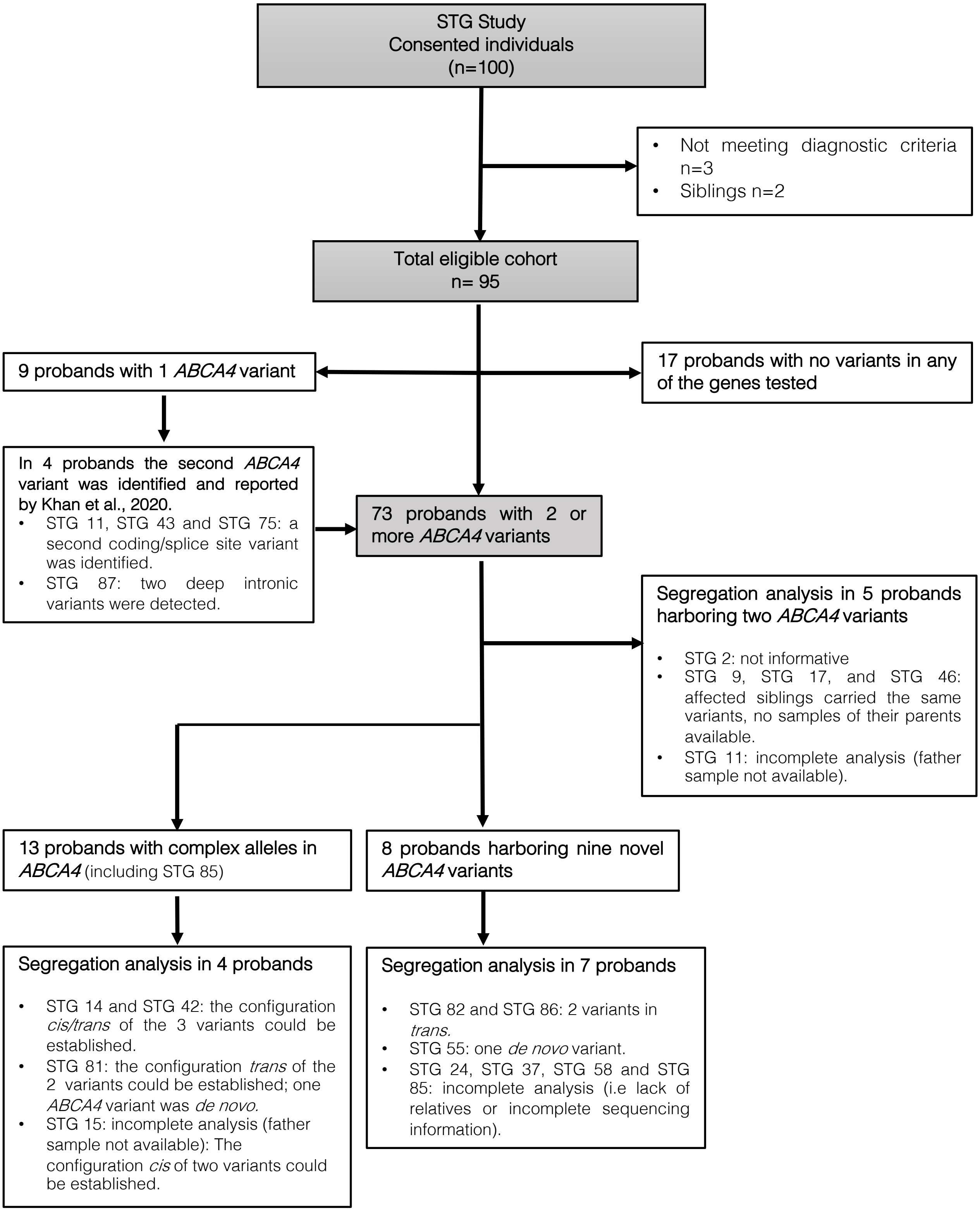

Supplementary Figure 1: Flow diagram showing the different stages of the study.

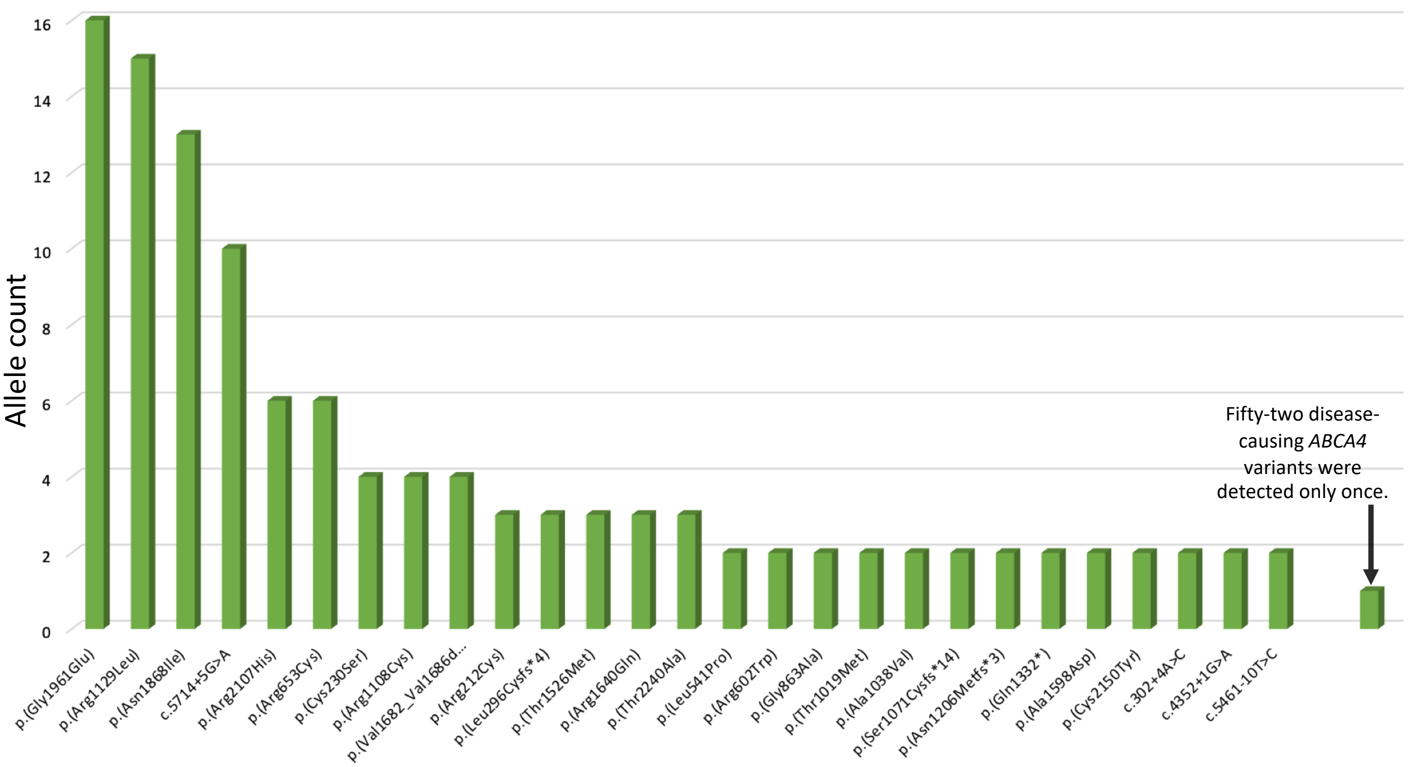

Supplementary Figure 2: *ABCA4* variant allele count in this study. Twenty-seven *ABCA4* variants were identified more than once. The disease-causing variants p.(Gly1961Glu) (n=16) and p.(Arg1129Leu) (n=15) were found most frequently, and p.(Leu541Pro) and p.(Ala1038Val) were only found as complex allele p.[Leu541Pro;Ala1038Val] (n=2). Most of the disease-causing *ABCA4* variants (n=52) were detected only once.
